# Foil Cylinders

**Published:** 1872-04

**Authors:** 


*Foil Cylinders.*—Dr. H. S. Chase in the *Missouri Dental Journal,* speaks as follows of Williams' prepared cylinders now for sale at the depots: "Williams, of New York, has introduced to the profession, gold cylinders of different sizes, made of soft foil, but which become adhesive by *heating.* These cylinders are intended for "cylinder filling" according to the usual manipulation of gold cylinders. For regular cylinder fillings, I prefer to make my cylinders of Abbey's foil, and wind them pretty *tightly,* so as to have as much gold as possible in a small diameter. I find Williams' cylinders are too loosely wound for my purpose in this direction ; on the other hand I am delighted to find that I can use them as *adhesive pellets.* I have made quite a number of contour plugs of them ; they adhere firmly and pack smoothly. They should be laid flat, so as to mallet against their sides, instead of their ends. Much time is saved in using them, as there is more gold in the same bulk than in a twisted pellet. They are cut in some peculiar way, so that their ends are not *condensed,* as when cut by scissors.

They are made of five different numbers. Number ½ is made by rolling up a half sheet of No. 4 gold foil. as small in diameter as No 17, of the wire gauge, and about three times as long.

No. 1 is made, by using in the same way, one whole sheet of No. 4 gold foil.

No. 2, by using in the same manner, two whole sheets, laid together.

No. 3, by using three sheets laid together.

No. 4, by using four sheets laid together.

The *diameter* of No. 1, is about thirteen of the gauge: that of No. 2, about 10 of the gauge ; that of No. 3, about 9 ; and No. 4, about 8 of the gauge. Their *lengths* vary in the same numbers.

This preparation of gold costs 25 cents more per ⅛ of an ounce, but as there is a saving in respect of *waste,* it is quite as economical, and, perhaps, more so than gold foil simply. It is put up in small boxes.

There now is quite a puff for these cylinders. but it is not a paid puff. I bought and paid for the ones I have used, and I have written this for the benefit of the profession, and not for Mr. Williams, though I heartily thank him for having made and put into market so *convenient* an article."

